# Supplementary material for: Unravelling reference bias in ancient DNA datasets
Source: Bioinformatics. 2024 Jul 3;40(7):btae436. doi: 10.1093/bioinformatics/btae436 (PMC11254355; doi:10.1093/bioinformatics/btae436)
Supplement: btae436_Supplementary_Data [file btae436_supplementary_data.zip › S1-S4_data_plots.zip!/S1-S4_data/mapping_stats_plots.zip!/mapping_stats_plots/verysensitive_bowtie_elephant_div0_50end_mapping_stats_byMQ_plots.pdf]

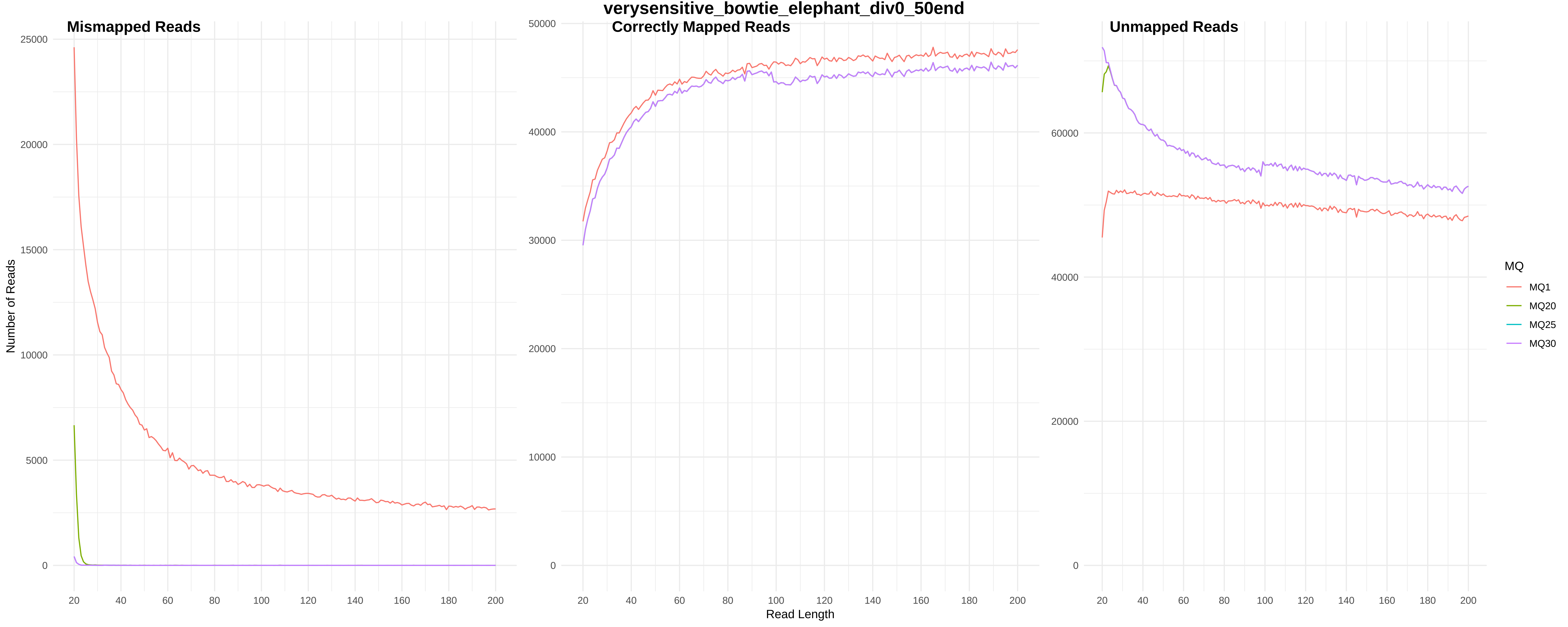
verysensitive_bowtie_elephant_div0_50end
Mismapped Reads
Correctly Mapped Reads
Unmapped Reads
Number of Reads
Read Length
25000
20000
15000
10000
5000
0
50000
40000
30000
20000
10000
60000
20 40 60 80 100 120 140 160 180 200
MQ
MQ1
MQ20
MQ25
MQ30
